# Attentional bias during emotional processing: Behavioral and electrophysiological evidence from an Emotional Flanker Task

**DOI:** 10.1371/journal.pone.0249407

**Published:** 2021-04-02

**Authors:** Natalia Trujillo, Diana Gómez, Sandra Trujillo, José David López, Agustín Ibáñez, Mario A. Parra

**Affiliations:** 1 Neuroscience Group, University of Antioquia UdeA, Medellín, Colombia; 2 GISAME, Facultad Nacional de Salud Pública, Universidad de Antioquia UdeA Medellín, Medellín, Colombia; 3 SISTEMIC, Facultad de Ingeniería, Universidad de Antioquia UdeA, Medellín, Colombia; 4 Global Brain Health Institute (GBHI), University of California San Francisco (UCSF), San Francisco, California, United States of America; 5 Trinity College Dublin (TCD), Dublin, Ireland; 6 Cognitive Neuroscience Center (CNC), Universidad de San Andrés, Buenos Aires, Argentina; 7 National Scientific and Technical Research Council (CONICET), Buenos Aires, Argentina; 8 Latin American Institute for Brain Health (BrainLat), Universidad Adolfo Ibanez, Santiago, Chile; 9 School of Psychological Sciences & Health, University of Strathclyde, Glasgow, United Kingdom; 10 Universidad Autónoma del Caribe, Barranquilla, Colombia; Universita degli Studi di Roma La Sapienza, ITALY

## Abstract

Threatening stimuli seem to capture attention more swiftly than neutral stimuli. This attention bias has been observed under different experimental conditions and with different types of stimuli. It remains unclear whether this adaptive behaviour reflects the function of automatic or controlled attention mechanisms. Additionally, the spatiotemporal dynamics of its neural correlates are largely unknown. The present study investigates these issues using an Emotional Flanker Task synchronized with EEG recordings. A group of 32 healthy participants saw response-relevant images (emotional scenes from IAPS or line drawings of objects) flanked by response-irrelevant distracters (i.e., emotional scenes flanked by line drawings or vice versa). We assessed behavioural and ERP responses drawn from four task conditions (Threat-Central, Neutral-Central, Threat-Peripheral, and Neutral-Peripheral) and subjected these responses to repeated-measures ANOVA models. When presented as response-relevant targets, threatening images attracted faster and more accurate responses. They did not affect response accuracy to targets when presented as response-irrelevant flankers. However, response times were significantly slower when threatening images flanked objects than when neutral images were shown as flankers. This result replicated the well-known Emotional Flanker Effect. Behavioural responses to response-relevant threatening targets were accompanied by significant modulations of ERP activity across all time-windows and regions of interest and displayed some meaningful correlations. The Emotional Flanker Effect was accompanied by a modulation over parietal and central-parietal regions within a time-window between 550-690ms. Such a modulation suggests that the attentional disruption to targets caused by response-irrelevant threatening flankers appears to reflect less neural resources available, which are seemingly drawn away by distracting threatening flankers. The observed spatiotemporal dynamics seem to concur with understanding of the important adaptive role attributed to threat-related attention bias.

## 1. Introduction

When attention is drawn by co-occurring threatening and non-threatening stimuli, it biases towards the former. This processing bias, which has been previously called the threat or anger superiority effect [1–3] is considered a fast-adaptive mechanism to avoid risks or danger [4, 5]. Such a response appears to enable a rapid discrimination of dangerous stimuli embedded in rich sensory environments [6, 7]. The threat-related attentional bias has been observed experimentally with a wide variety of tasks and stimuli [1, 8–13]. Yet, the spatiotemporal dynamics of the neurocognitive mechanisms underpinning this adaptive response remain unclear.

Using fMRI, Cisler & Koster [14] showed that early visual processing of threatening stimuli was supported by activation over occipital-parietal areas and the amygdala, while engagement/disengagement of attention has been associated with activation of the prefrontal cortex. Behavioral studies have indicated that the disengagement of attention from threatening faces requires more time and executive resources than from non-threatening faces [15, 16]. These findings have been supported by studies using Event Related Potentials (ERP). Modulations of early (e.g., N200, N2pc, P1, N1) as well as late ERP components (e.g., P3, LPP) have been reported during threat-related attentional processing [9, 17–21]. A recent ERP study which used real-life scenes from the International Affective Picture System (IAPS) and faces suggested that processing threats triggers early posterior visual components (around 170 ms for faces and 220–230 ms for IAPS images) and may lead to later activation of anterior attentional mechanisms [22]. Taken together, these results suggest that the threat-related attentional bias may reflect the orchestrated dynamic of different attentional mechanisms and that an ERP analysis may contribute valuable information about its neural correlates [23].

From a behavioural perspective, a task derived from Biased-Competition Models seems appropriate to investigate the interplay of attentional mechanisms involved in the attentional bias to threats [16, 24, 25]. This model proposes that stimuli compete for limited resources responsible for selecting the most relevant information that can hold adaptive value [26]. The competition biases attention towards salient information, as relevant stimuli are selected for later analysis and multidimensional integration (i.e. space, time, location) [26]. By presenting two simultaneous stimuli and therefore in direct competition, such tasks can provide evidence of attentional bias towards response relevant or irrelevant information [13, 16]. The methodology used by the Flanker Task [27] seems appropriate to investigate such hypotheses [28, 29]. This task is sensitive to evaluate attentional mechanisms (e.g. orienting, engagement/disengagement, conflict monitoring) [30] and it has been used to investigate the threat-related attentional bias [6, 25]. Threatening flankers would elicit activation of early exogenous attentional processes (i.e., orienting attention towards salient information [31], while targets would trigger late endogenous processes (i.e., engagement and disengagement, conflict monitoring). Therefore, such a task promotes competition and in doing so, it can help unveiling the neural sources of attentional bias.

Such a proposal has been recently supported by a study designed to address this hypothesis [27]. Parra et al. [27] demonstrated the constancy of the threat-related attentional bias in three experiments that manipulated temporal and perceptual aspects of the task. They consisted of varying the stimulus presentation time and presenting IAPS images as targets and flankers (Experiment 1 and 2), and IAPS images and objects as targets and flankers (Experiment 3). The authors investigated if the nature of the information competing for attention could play a role. In all the experiments the authors observed the threat-related attentional bias. However, reducing perceptual interference by making the stimuli competing for attention more distinct (Experiment 3) allowed assessing the threat-related attentional bias under less conflicting conditions, therefore demonstrating the existence of this response beyond the classical congruency paradigms (IAPS images vs IAPS images). Following this observation, we chose Parra et al.’s [27] Experiment 3 for the present study, as we are interested in the threat-related attentional bias uncontaminated by congruency effects. Using the Emotional Flanker Task, Parra and collaborators [27] demonstrated that when emotional and non-emotional stimuli compete for attention, the former attracts more resources (yielding faster responses) when presented as a response-relevant target and slows-down responses to non-IAPS targets (i.e., objects) when presented as a response-irrelevant flanker. Although this evidence provides support to the above-mentioned hypothesis, it still holds limited value to unveil the temporal dynamics supporting the attentional mechanisms associated to the threat-related attentional bias. Combined with the ERP analysis, the Emotional Flanker Task may provide a more reliable approach to investigate such contributions.

The spatiotemporal dynamics of the neurocognitive mechanisms underpinning this adaptive response are still not well understood. For instance Carretié, 2014 [32] could not include such studies in a meta-analysis of the relevant literature because they did not reach the 8-study threshold set for such analysis. We reviewed the literature produced over the last 10 years searching for studies that relied on the use of EEG-ERP tools and incorporated paradigms that allow: i) assessing Biased-Competition Models of attention during emotional stimuli processing under congruent/incongruent stimuli presentation; ii) exploring the influence of overt/covert attention mechanisms, and iii) included EEG/ERP analysis. Out of 32 publications produced in this period reporting on attention bias to threat (see S1 Table in S1 File for a detail report of the outcomes form the literature review), we found that 25 reported on outcomes that can advance our understanding of Biased-Competition Models. Of these, 14 investigated the interplay of covert/overt attention mechanisms and only three informed about congruency effects.

Publications contributing evidence on covert/overt attention mechanisms, which are the most relevant to the present study, reported the use of a variety of stimuli including IAPS Images [33–38], Chinese proverbs [37], other affective pictures like sports or body expressions [39, 40], arousing or aversive sounds with images [13, 41, 42], manipulated photos of participants [43], or faces [44, 45]. As it had been previously noted by [32], the majority of the reviewed studies found evidence of greater exogenous attention to emotional than to neutral distractors. Common to all these studies, modulations of early physiological activity (e.g., steady-state visual evoked potentials, P1, P2) underpinned more exogenously driven attention functions, while late modulations (e.g. LPP) were informative of more endogenous control of attention, and this was true regardless of the stimuli nature. Only one of these studies [44] used a task that resembled the flanker paradigm. The other studies investigated competition for attention relying on aspects of the same stimuli (foreground vs. background), overlapped images, co-occurring images and sounds, or different attention functions (e.g., Stroop inhibition). As we argued above, the Emotional Flaker Task presents two simultaneous stimuli which allow direct competition in conditions like those we encounter in real life (e.g., while navigating in crowded spaces). To our knowledge, such a task has not been used before to investigate the neurocognitive underpinning of the attention bias to threat. Furthermore, Carretié, 2014 [32] suggested that behavioural paradigms that explore such an attention bias by means of tasks that entail non-perceptual competition (e.g., object categorization as in our current task) are necessary. Considering the scarcity and variability across relevant studies encountered in the literature, we anticipate that this task would shed new light on the role of controlled and automatic attention mechanisms in the attention bias to threat and their neurocognitive drivers.

We followed a component-free approach to unveil the neural correlates of this effect. This approach avoids selection biases of time-windows and electrodes from electroencephalographic (EEG) recordings when contrasting the experimental conditions. Such an approach has been recommended for new tasks or tasks that have not been previously subjected to ERP studies [46]. It allows identifying relevant spatiotemporal aspects of the ERP activity linked to the task avoiding both selection bias and Type I error (i.e., via permutation analyses [47]). We aim to provide behavioural and electrophysiological evidence to support our hypothesis that the threat-related attentional bias found with the Emotional Flanker Task can be accounted for by a competition of attentional mechanisms operating at different spatiotemporal dynamics. Based on previous EEG-ERP studies which have investigated similar hypotheses [21], we predict that an automatic (i.e., exogenous) component of the threat-related bias would be likely linked to early ERP modulations. For instance, activity occurring in the rage of the P1 component could be associated with the process of orienting attention [48]. Such an early activity has been reported over parietal-occipital electrodes [49]. In the context of the Emotional Flanker Task, P1-like modulations may reveal early influences of stimulus saliency on responses to targets with threating flankers, which may lead to an attentional disengagement from the target [16]. Such an interference would have behavioural implications (i.e., the flanker effect).

The influence of other attention orienting functions such as shifting and engagement, which are necessary to monitor conflict and allocate attentional resources [16], would likely emerge during later time windows as these would be driven by top-down mechanisms (i.e., endogenous). Previous ERP studies have reported that N2 and LPP components elicited over anterior regions (i.e., frontal-central) are associated with such attention functions [50, 51]. In the context of the Emotional Flaker Task, modulations occurring during a time window compatible with N2 would likely index identification of mismatches between threatening and neutral targets and flankers competing for attention [52]. Later modulations (e.g., LPP) would support functions such as goal maintenance, inhibitory control [53], integration of contextual information, and emotional indexing [54], which are under more influence of top-down control mechanisms driven by frontal regions.

We predict that if the effect induced by Threatening Flankers does reflect interference occurring during the orienting stages of disengaging attention, early ERP modulation should be observed over the posterior attentional network [50, 55]. Moreover, we predict that if the responses to central targets do reflect the function linked to shifting and engaging attention, late ERP modulations should be observed [51, 56].

## 2. Methods

### 2.1. Participants

Thirty-two healthy undergraduate students participated in the experiment (17 men, 15 women). Their mean age was 20.2 years old (SD = 2.7) with a mean educational level of 13.9 years (SD = 2.3). This paper builds upon data from the research project: “Emotional processing and its modulation in people reinserted from the Colombian armed conflict, belonging to the high council of the presidency of the Republic of Colombia, Antioquia region” approved by the Ethics Committee of the research unit of the University of Antioquia (File No. 09-010-213, April 16, 2009). This study was conducted in accordance with the ethical standards stated in the Declaration of Helsinki [57]. All participants signed the informed consent approved by the Ethics Committee of the University of Antioquia. The participants were free of psychiatric or neurological disorders and did not report use of psychoactive substances.

### 2.2 Assessment

#### 2.2.2 The Emotional Flanker Task

This task is the one reported in Parra et al. [27] (Experiment 3), which was adapted from the model originally proposed by Eriksen and Eriksen [58] and Horstmann et al., [25]. The task was programed in E-Prime (Psychology Software Tools, Pittsburg, USA). The key conditions of the Emotional Flanker Task are defined based on the content and position of emotionally relevant stimuli. Line drawings (i.e., objects) are used to pose competition for attention without overlap in the emotional dimension. The rationale for this selection was previously explained by Parra et al. [27] and outlined in the Introduction. From this perspective, our design includes: 1) Neutral images as Target and Flanker: 1.A - Target Neutral/Flanker Object, 1.B - Target Object/Flanker Neutral; 2) Threat as Target and Flanker: 2.A - Target Threat/Flanker Object, 2.B - Target Object/Flanker Threat. Note that the Emotional Flanker Task used in the present study does not address congruency effects, as Targets and Flankers were always from different dimensions (IAPS images or Objects).

**Fig 1:**
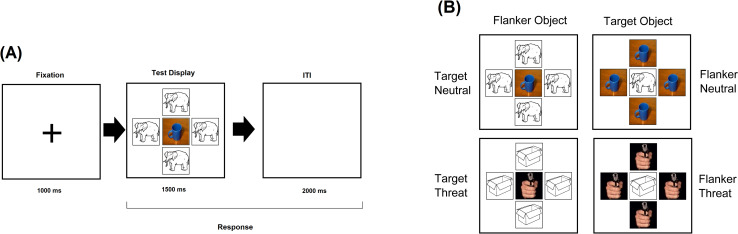
Emotional Flanker Task design. **(A)** Trial sequence of the Emotional Flanker Task. **(B)** Examples of test display used in each experimental condition. *Note*. We used the affective ratings of IAPS images [62] and for the objects the International Picture Naming Project database [61] to select the stimuli for this task.

The task did not include trials where Target and Flanker could be both either IAPS images or Objects (see Parra et al. [27]–Experiments 1 and 2– for a version of the task that assesses the Congruency effect). When the target was an IAPS image the Flankers were Objects, and vice versa. Sixty threatening images and sixty neutral images were chosen from the IAPS normative database using the valence and arousal provided by Lang et al. [59] (threatening stimuli: Valence M = 2.7, SD = 0.7, Arousal M = 6.0, SD = 0.8; neutral stimuli: Valence M = 5.9, SD = 1.1, Arousal M = 3.4, SD = 0.9). According to Bradley & Lang 9-point scale [60], valence scores for negative images are low (e.g., < 4) and increase for neutral and positive images. In addition to the IAPS images, thirty living (e.g., cat) and thirty non-living (e.g., broom) objects were selected from the International Picture Naming Project database [61], all with naming frequency above 80%. The experiment was divided in 4 blocks, each consisting of 60 trials using the layout presented in Fig 1 (240 trials in total). IAPS images belonging to each valence were presented twice, one as targets and one as flankers. Living and non-living images were presented four times each, flanking neutral and threating IAPS images, and flanked by neutral and threatening IAPS images [62]. The trial sequence is shown in Fig 1. The duration of the task was approximately 20 minutes.

At the beginning of each trial, a fixation cross was presented for 1000 ms, followed by a test display presented for 1500 ms. Participants were requested to press one of two previously allocated keys of a standard keyboard as quickly and accurately as possible depending on whether the central image showed a “Neutral Scene/Non-living Object” (key “v” with a green sticker) or a “Threatening Scene/Living Object” (key “n” with a red sticker). There was then an inter-trial interval of 2000 ms during which responses were still recorded (see Fig 1).

As our key hypotheses concerned effects driven by real-life emotional images (i.e., IAPS), we followed a 2x2 design: at Central Position–Threat/Neutral as Target (TC-2.A/NC-1.A) and at Peripheral Position–Threat/Neutral as Flanker (TP-2.B/NP-1.B). The living/non-living categories of objects were response irrelevant and were only used to add attentional demands and increase competition. This explains why we did not dichotomize the objects but only the emotional dimension (i.e., neutral vs threat). In fact, Parra et al. [27] reported that the effect of Object Category (i.e. Living vs. non-Living) proved non-significant [F (1, 28) = 1.34, p = .256; η^2^ = 0.21, β = 0.20] and had no impact on the key interaction reported. That led them to collapse responses across these stimuli and refer to them as “Object”. We followed the same approach. Trials and blocks were fully randomized across participants. We computed accuracy and response time when threatening and neutral stimuli were presented either as Targets or as Flankers.

### 2.3. EEG recording

The Flanker task was synchronized with EEG recording. A 64 electrodes NeuroScan Ltd. system with a 10–10 configuration easy-cap was used for acquisition. The signal was recorded with the software Scan 4.5 using a sampling rate of 1000 Hz. Impedance was kept below 10 kΩ. The assessment was conducted within a Faraday chamber with dimmed lights. Participants were seated 60 cm away from a 17” standard PC monitor where stimuli were presented. The total duration of the testing session was approximately 1 hour.

### 2.4. Testing procedures

The assessment was carried out in a single session. After completing the Emotional Flanker Task, we carried out an emotional screening procedure using a task that asked participants to rate in the two evaluated dimensions (threat and neutral) each IAPS image previously seen using a 5-point Likert scale. This procedure was undertaken to ensure that the perceived valence matched the one reported by Lang et al. [59].

### 2.5. Signal processing

**Fig 2:**
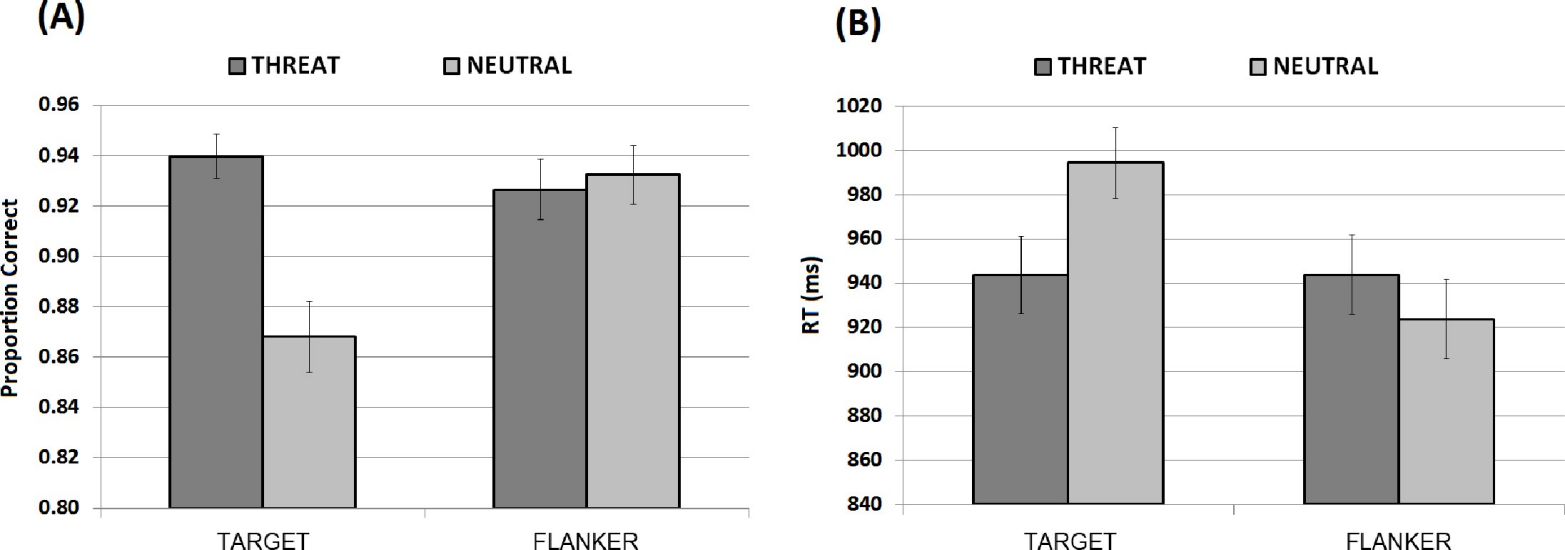
Accuracy and reaction time of Flanker Task. Mean accuracy data **(A)** and response time **(B)** across the two experimental factors, Emotion-type and Emotion-position (Error bars represent 95% CI; * = p < 0.05).

EEG data were analysed with the toolbox EEGLAB version 2019–0 under Matlab 2019a. The data were downsampled to 250 Hz and filtered with a high-pass filter of 0.1 Hz. It was then re-referenced from mastoid electrodes to the average. Next, we performed an independent component analysis (ICA) as implemented in EEGLAB. We rejected components informing high frontopolar activity, which are associated with oculomotor artifacts; as well as other non-ocular signals based on neurophysiological criteria. Following recommendations by Luck [63], we eliminated slow drifts and high-frequency noise prior to running ICA. We used *runica* (it calls runica.m) and jader (it calls the function jader.m) which are a part of the default EEGLAB. The maximum number of rejected independent components was 5%. A maximum of two trials per condition were discarded during further visual artefact rejection after ICA. Then, we applied a low-pass filter at 30 Hz and epoched the data. Blocks were epoched (Threat as Targets, Threat as Flankers, Neutral as Targets, and Neutral as Flankers) with a time window between -200 ms pre- and 800 ms after-stimuli presentation. A baseline correction using the -200 to 0 ms window was applied. Only correct trials entered the ERP analysis. The average number of trials useful for analysis was always above 80% (see Fig 2 for accuracy data).

### 2.6. ERP analysis

The Emotional Flanker Task has not been previously used in ERP studies. This would make it difficult to rely on specific ERP components to investigate the electrophysiological correlates of the attentional bias to threat found with such a test. As Kappenman and Luck [64] acknowledge, using the same time windows from previous studies can be difficult for experiments involving new tasks or experimental manipulations, or for experiments involving populations or age groups that have not been studied in the same context. An approach that the authors suggested to deal with both the selection of time windows and regions of interest (ROIs) is to perform separate statistical tests for each time point at each electrode, combined with a correction for multiple comparisons [65]. We followed this approach.

With this approach, we searched for significant differences across conditions with a combination of the Monte Carlo test and non-parametric bootstrapping running 1000 permutations, using the implementation available at the EEGLAB toolbox: *statcond*(). We focused on the key contrasts which have previously revealed the Flaker Effect [27] (Threatening Target vs Neutral Target / Threatening Flanker vs Neutral Flanker). Then, we generated a Monte-Carlo approximation histogram of the permutation distribution and compared it with the estimated permutation p-value, which is the proportion of random partitions where the observed test statistic is larger than the value from the permutation distribution. Data with a p-value smaller than the selected critical alpha-level (0.01) were considered significantly different.

Permutations were calculated following a component-free approach across the entire array of electrodes for every datapoint. However, a problem of using such permutations is that they show every separation between both signals, which is not necessarily an effect (commonly known as overfitting). To avoid this, we set some criteria for selecting time windows and ROIs: a) we ignored windows where consecutive significant differences lasted less than 40ms, as most of its activity could be spurious (again, overfitting); b) in active (statistically significant) regions with several electrodes, we selected the ones with time windows meeting the above criterion. Removed electrodes may be considered boundaries of the activity and not directly relevant; and c) we ignored activity of single electrodes if no neighbour showed the same activity, as it was considered correlated to larger ROIs at the same window.

Following this procedure, we formed four ROIs: i) parietal and centro-parietal regions (P1, P2, P3 and PZ, CP1, CP2, CP3 and CPZ) with significant differences appearing within 300–400 ms (our earliest significant time window); ii) parietal and central-parietal regions (P1, P3 and PZ, CP3, CP1 and CPZ) with significant differences appearing within a late time window (550–690 ms); iii) parietal and parietal-occipital regions (P1, P3, and P5, PO3, PO5, PO7 and POZ) with significant differences appearing also within a late time window (500–700 ms); and iv) frontal regions (FP1, FP2 and FPZ) with significant differences appearing within a late time window (500–700 ms).

### 2.7. Statistical analysis

Parra et al. [27] previously reported that the Object Category (i.e. Living vs. non-Living) did not drive significant effects (see description above). We therefore followed the same methodological approach proposed by these authors and collapsed responses across these stimuli and referred to them as “Object” in the following analysis. We implemented a two-way repeated-measures ANOVA model. The first repeated measure was Emotion position (2 levels: Target vs. Flanker) and the second was Emotion type (2 levels: Neutral or Threatening) (see Fig 1 for more details of the task design). The interaction between Emotion position and Emotion type enables investigating the impact of the identity of images presented as Flankers on the responses to images presented as Targets when these images hold different emotional properties. For main effects and the interaction, we report effect size as informed by eta-square (ƞ^2^) (0.1 = small, 0.24 = medium, and 0.31 large) and power (β). To further investigate significant main effects and interactions, Bonferroni corrected post-hoc analyses were carried out using paired sample t-tests. For post-hoc analyses the effect size was calculated using Cohen-*d* (0.2 = small, 0.5 = medium, and 0.8 large).

For the ERP analysis, we selected the average activity for each of the four ROI and their corresponding time windows identified following a component-free approach. Such an approach allowed us to identify the spatiotemporal distribution of significant differences among the experimental conditions that reveal the attention bias to threat, namely Target Threatening vs Target Neutral and Flanker Threatening vs Flanker Neutral. To explore if such electrophysiological responses dissociated across the two experimental factors: Emotion position and Emotion type, we subjected these data to the same ANOVA models described above for the behavioural responses. Unless otherwise stated, the assumption of homogeneity of variance was met. The statistical threshold was set to p = 0.05. Additionally, we checked the number of ERP trials used for the analysis of each condition and performed a chi-square (X^2^) analysis to identify differences. We failed to find significant differences in the frequency distribution of trials across the four experimental conditions (see, S2 Table in S1 File). Finally, we performed a Pearson correlation analysis to identify if there was any association between behavioural variables and ERPs for each condition (see, S3–S6 Tables in S1 File).

## 3. Results

### 3.1. Behavioral data from Emotional Flanker Task

#### 3.1.1. Accuracy

The repeated-measures ANOVA model revealed a main effect of Emotion type [*F*(1,31) = 12.34, p < 0.001, ƞ^2^ = 0.53, β = 0.93]. Threatening images received more correct responses than Neutral Images [*t* = 3.51, p = 0.001, *d* = 0.47]. Emotion position also yielded a significant effect [*F*(1,31) = 5.63, p = 0.02, ƞ^2^ = 0.39, β = 0.63]. Target objects (with flanking IAPS images) attracted more correct responses than Target IAPS images (with flanking objects) [*t* = 2.37, p = 0.024, *d* = 0.39]. The Emotion type x Emotion position interaction was significant [*F*(1,31) = 21.20, p < 0.001, ƞ^2^ = 0.64, β = 0.99]. This reflected an emotion-related discrepancy when IAPS images were presented as targets, whereby Threatening images attracted more correct responses than Neutral images [*t* = 4.46, p<0.001, *d* = 0.82]. Such a discrepancy was not observed when IAPS images were presented as flankers [*t* = 0.78, p = n.s., *d* = 0.07].

#### 3.1.2. Response time

The repeated-measures ANOVA model revealed a main effect of Emotion type [*F*(1,31) = 4.56, p = 0.04, ƞ^2^ = 0.36, β = 0.54]. Threatening images yielded faster responses than Neutral Images [*t* = 2.14, p = 0.04, *d* = 0.14]. Emotion position also yielded a significant effect [*F*(1,31) = 14.67, p < 0.001, ƞ^2^ = 0.57, β = 0.96]. Target objects (with flanking IAPS images) attracted faster responses than target IAPS images (with flanking objects) [*t* = 3.83, p = 0.001, *d* = 0.31]. Crucially, the Emotion type x Emotion position interaction was significant [*F*(1,31) = 28.57, p < 0.001, ƞ^2^ = 0.69, β = 0.99]. This indicates that Threatening images presented as Targets were identified faster than Neutral images [*t* = 4.11, p<0.001, *d* = 0.45]. However, when Threatening images were presented as flankers, they slowed the detection of Target objects more significantly than when Flankers were Neutral images [*t* = 3.24, p = 0.003, *d* = 0.16].

In summary, images with threatening content were detected more accurately and faster than Neutral images when they appeared as targets. Emotion does not tend to differentially affect accuracy when IAPS images flank non-emotional targets (i.e., objects). However, emotion does differentially affect response times when IAPS images flank object targets; whereby threatening but not neural images significantly reduced the speed at which such targets are detected.

It has been suggested that brightness differences of IAPS images can influence the evaluation of affective pictures [66]. To rule out the contribution of brightness to the effects described above, we ran an additional ANCOVA analysis in which we entered the luminance of the IAPS images used in the Emotional Flanker Task as a covariate. We present these results in Table 1. Luminance differed across emotional valences (i.e., Threatening images were darker than Neutral images as reported previsouly). However, luminance explained neither the discrepancy yielded by Threat Flanker–Neutral Flanker nor the one observed with Threat Target–Neutral Target. Such discrepancies drove the significant Emotion type x Emotion position interaction described above. Moreover, the emotional screening task performed after the Emotional Flanker Task confirmed that the perceived valence of IAPS images matched that reported by Lang et al. [59] (see S7 Table in S1 File). Therefore, perceptual factors cannot be the drivers of the effects described above.

**Table 1:** Effects of IAPS images luminance on the discrepancies yielding the significant interaction reported in the behavioral analysis of response time data.

		**Mean**	**SD**	***t***	**p-value**
Luminance Threatening		117.4	30.4	24.79	<0.001
Luminance Neutral		90.2	35.0		
	** **	**Response Time**		**Accuracy**	
**Discrepancy**	** **	***F***	**p-value**	***F***	**p-value**
TC-NC	Luminance	3.91	0.048	6.06	0.010
	Emotion	39.20	**<0.001**	50.39	**<0.001**
TP-NP	Luminance	2.16	0.142	0.00	0.980
	Emotion	6.73	**0.010**	0.35	0.553

*Note*. Luminance was calculated using Matlab (see: https://www.mathworks.com/help/matlab/ref/rgb2gray.html#buiz8mj-9).

### 3.2. ERP data from the Emotional Flanker Task

#### 3.2.1. ROI-1 (parietal and central-parietal regions) in a time window (300 – 400ms)

The repeated-measures ANOVA model revealed non-significant effects of Emotion type [*F*(1.31) = 3.33, p = 0.08, ƞ^2^ = 0.10, β = 0.42] or Emotion position [*F*(1,31) = 1.79, p = 0.19, ƞ^2^ = 0.05, β = 0.25]. The Emotion type did significantly interact with Emotion position [*F*(1.31) = 6.08, p = 0.02, ƞ^2^ = 1.16, β = 0.67]. A *post hoc* analysis shows that threatening images as targets yielded larger positive amplitudes than neutral images as targets [*t* = 2.4, p = 0.02, *d* = 0.49]. Such a discrepancy was not observed when images were presented as Flankers [*t* = -0.33, p = 0.74, *d* = -0.05].

#### 3.2.2. ROI-2 (parietal and central-parietal regions) in a time window (550 – 690ms)

The repeated-measures ANOVA model revealed a non-significant main effect of Emotion type [*F*(1.31) = 2.71, p = 0.11, ƞ^2^ = 0.08, β = 0.36] or Emotion position [*F*(1.31) = 0.21, p = 0.65, ƞ^2^ = 0.01, β = 0.07]. The Emotion type x Emotion position interaction was significant [*F*(1.31) = 14.68, p = 0.00, ƞ^2^ = 0.32, β = 0.96]. A *post hoc* analysis shows that threatening images as targets yielded larger negative amplitudes than neutral images as targets [*t* = 3.3, p = 0.00, *d* = 0.59] with the inverted pattern observed when images were presented as flankers (neutral images elicited larger negative responses than threatening images; *t* = -2.8, p = 0.01, *d* = -0.29).

#### 3.2.3. ROI-3 (parietal and parietal-occipital regions) in a time window (500 – 700ms)

The repeated-measures ANOVA model revealed significant main effects of Emotion type [*F*(1.31) = 6.47, p = 0.02, ƞ^2^ = 0.17, β = 0.69] and Emotion position [*F*(1.31) = 12.84, p = 0.00, ƞ^2^ = 0.29, β = 0.93]. The Emotion type x Emotion position interaction was significant [*F*(1.31) = 12.00, p = 0.00, ƞ^2^ = 0.28, β = 0.92]. A *post hoc* analysis shows that threatening images as targets yielded larger negative amplitudes than neutral images as targets [*t* = 3.2, p = 0.00, *d* = 0.54]. Such an effect was not found for images presented as flankers [*t* = -1.79, p = 0.08, *d* = -0.12].

#### 3.2.4. ROI-4 (frontal regions) in a time window (500 – 700ms)

**Fig 3:**
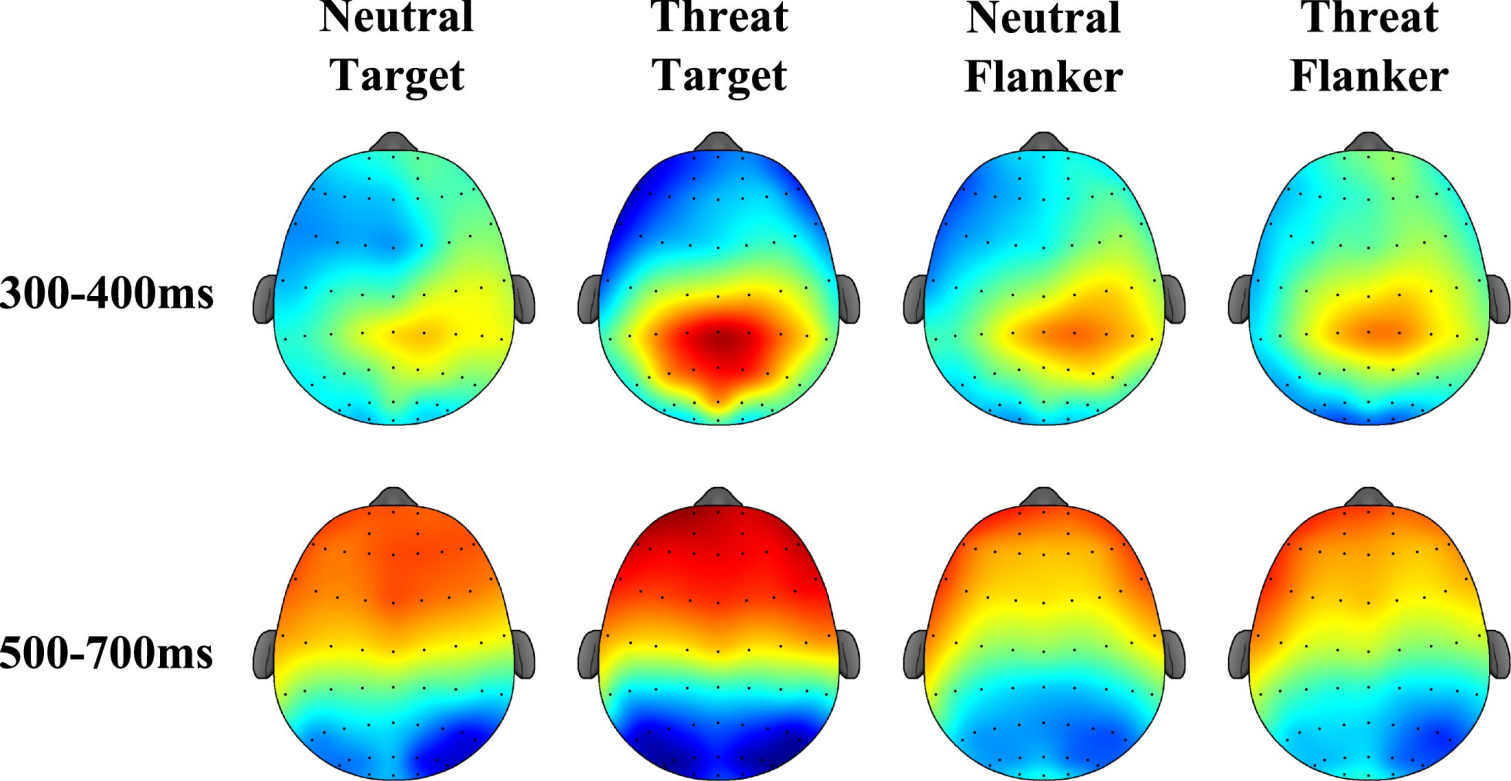
Scalp distribution during the Emotional Flanker Task. ERP activity observed around 300-400ms and 500-700ms across conditions.

**Fig 4:**
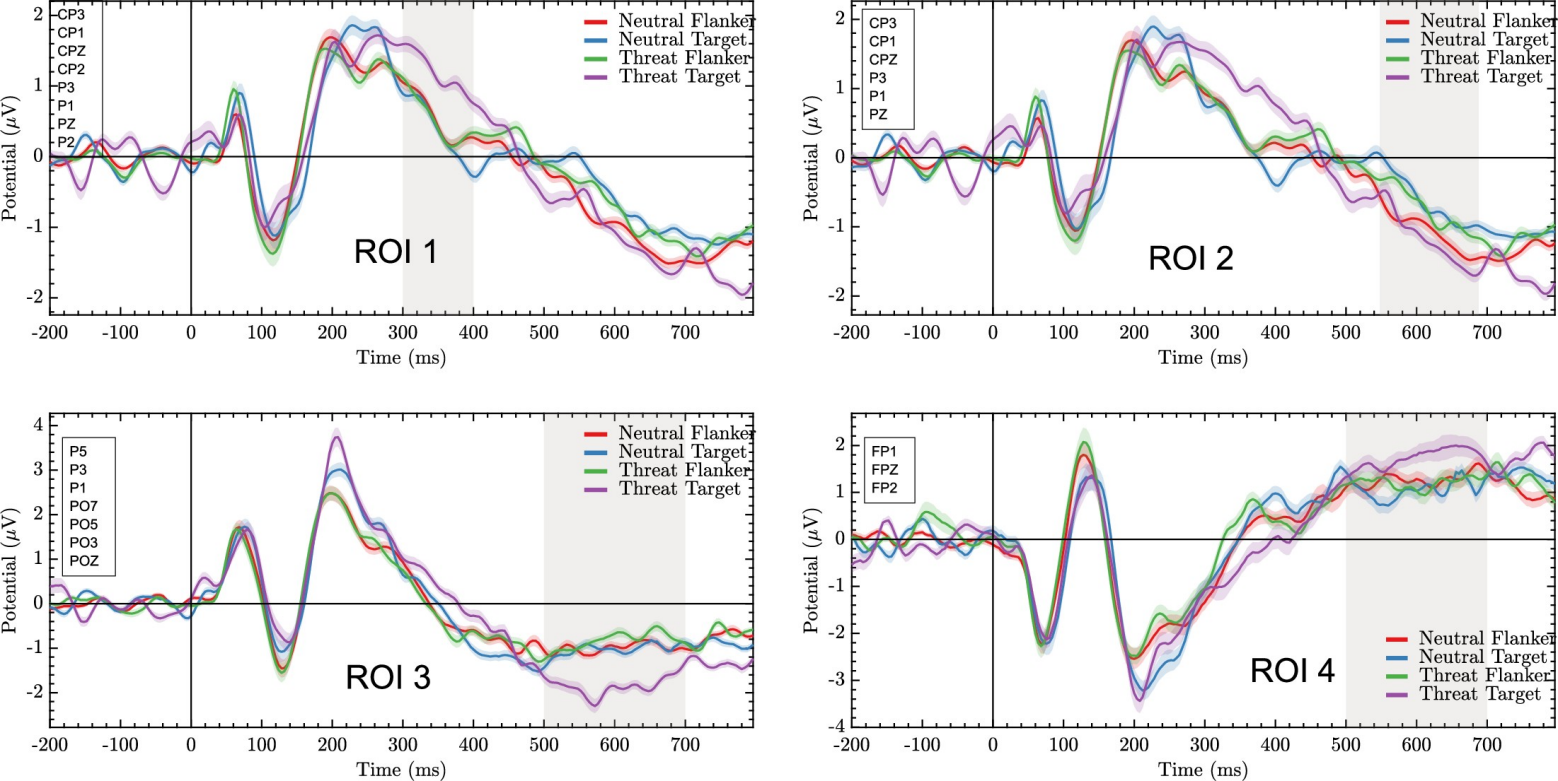
ERP recorded during the Emotional Flanker Task. Parietal and parietal-central early modulation **(ROI1)**; parietal and parietal-central intermediate modulation **(ROI2)**; parietal and parietal-occipital **(ROI3)** and frontal **(ROI4)** late modulation for Threatening and Neutral Emotion type as Target and Emotion position as Flanker.

The repeated-measures ANOVA model revealed a non-significant main effect of Emotion type [*F*(1.31) = 3.09, p = 0.09, ƞ^2^ = 0.09, β = 0.40] or Emotion position [*F*(1.31) = 1.40, p = 0.25, ƞ^2^ = 0.04, β = 0.21]. The Emotion type x Emotion position interaction was significant [*F*(1.31) = 6.47, p = 0.02, ƞ^2^ = 0.17, β = 0.69]. The *post hoc* analysis shows that threatening images as targets yielded larger amplitudes than neutral images as targets [*t* = 2.5, p = 0.02, *d* = 0.51]. The contrast between images presented as flankers was non-significant [*t* = -0.96, p = 0.34, *d* = -0.12]. In Fig 3 we show scalp distribution observed around 300-400ms and 500-700ms across conditions, and in Fig 4 ERPs recorded in the four ROIs.

Finally, a correlation analysis informed that the amplitude of the activity elicited over parietal and central-parietal regions in the 300 – 400ms time window significantly correlated with reaction time data from the Threat-Central condition, whereby larger amplitudes were associated to slower RT (r = 0.49, p = 0.00) (see S3 Table in S1 File). No other associations were identified (for the full set of correlations see S3–S6 Tables in S1 File).

In summary, these ERP analyses revealed a discrepancy for Threatening images presented as target relative to Neutral images presented as target, which was evidenced over extended brain regions and time windows (all ROIs and time windows that proved significant in our component-free approach). Interestingly, a pattern compatible with the behavioural dissociation previously reported using the flanker task was only found over parietal and central-parietal regions in a late time window (550 – 690ms). Our results also reveal that electrophysiological activity correlates with behavioural responses seemingly linked to endogenous attention resources deployed to process response relevant stimuli.

## 4. Discussion

The aim of the present study was to investigate whether combined behavioural and ERP evidence would shed new light on the spatiotemporal dynamics of the threat-related attentional bias observed with the Emotional Flanker Task. The literature review undertaken to support the rationale of this study confirmed the relevance of further exploring hypotheses linked to Biased-Competition Models of attention combining behavioural responses drawn from tasks akin to the flanker paradigm with the analysis of electrophysiological responses. Our key findings were: i) Threatening images presented as targets were detected faster and more accurately than Neutral images; however, the same images presented as flankers significantly slowed responses to targets; ii) The behavioural discrepancy observed for responses to Threatening Targets relative to Neutral Targets was accompanied by significant ERP discrepancies over extended brain regions involving frontal, central, parietal, and occipital regions, which expanded from 300ms to 700ms; iii) Contrary to our predictions, ERP discrepancies for Threatening Flankers relative to Neutral Flankers were only observed over parietal and central-parietal regions in a late time window. We now discuss each of these findings.

The flanker effect reported by Horstmann et al. [24] relying on the methodology devised by Ericksen and Ericksen [39] is linked to the emotional saliency of threat-related flankers, which increases the response time to stimuli presented as targets [3, 67]. Here, we corroborated such an effect with an Emotional Flanker Task [27] and sought evidence of the neural underpinnings of such a threat-related attentional bias. The behavioural results suggest that the attentional bias elicited by the Emotional Flanker Task may reflect competitive interaction between automatic (posterior) and controlled (anterior) attentional mechanisms. According with Pessoa et al. [68], flanker images are processed with an independent visual-spatial network if the instructions of the task emphasize on the competition. Zhou and Liu [69] proposed that emotion processing can be influenced by both controlled and automatic attentional mechanisms. The saliency of emotional information can render the attentional orienting process less reliant on top-down modulation and more driven by perceptual features of images appearing in the visual field. As we discuss below, our behavioural and electrophysiological data seem to be partially in line with this suggestion. Parra et al. [27] showed that the attentional bias found with the Emotional Flanker Task is apparent during both subliminal and supraliminal stimuli presentation. Using supraliminal stimulus presentation, here we replicated these findings and demonstrated that the saliency of threat-related response-irrelevant stimuli suffices to interfere with controlled attention mechanisms supporting the processing of response-relevant targets.

Additionally, we found faster and more accurate responses during Threatening Target trials [3, 15, 67–71] and significant modulations of ERP activity over frontal, central, parietal, and occipital regions. These findings support the controlled nature of the mechanisms sub-serving responses to response-relevant targets. These modulations spanned over the entire segment of the time window, which revealed significant differences across key experimental conditions; thus, suggesting a wide recruitment of neural resources to support such functions. Moreover, a significant correlation between behavioural and electrophysiological activity for the Threat-Central condition was found. It indicates that larger amplitudes in the intermediate stages of the information processing (intermediate ERPs) are accompanied by slower behavioural responses, seemingly denoting a more in-depth analysis of incoming stimuli. A similar association has been described by Bar-Haim et al., 2005 [72]. The authors interpreted this effect as an increased attention to threatening stimuli, rather than a general slow-down in execution. This suggests that the Threat-Central images capture higher attentional resources in the presence of competitive distracters.

Controlled activity seems to be also underpinned by late ERP activity widely spreading from central and central parietal regions (as denoted by activations found during the late time window over these regions) to frontal and parietal-occipital regions. Activity occurring within a similar time window (300–700ms, e.g., LPP component) has been linked to the encoding of emotional and motivational aspects of images in rich sensory contexts [73–75] and more specifically, to the local attentional bias induced by appetitive motivation [76]. In the context of this study, the ERP activity showed significant modulations for Threatening Targets relative to Neutral Targets during the two late time windows. This evidence leads us to suggest that within these time windows, the attentional system engages a wider network to perform a more in-depth analysis of the stimulus to confirm the presence of threats and to generate the corresponding response [77]. The fact that such activity recruited a more extended network involving occipital, parietal, central, and frontal regions is in line with this view (see also Carboni et al., 2017 [78]). This seems to be indexed by activity elicited over parietal and central-parietal regions within our earlier significant time window (300–400ms). Previous studies have reported that early ERP activity appears to index controlled processes responsible for identifying mismatches between targets and contextual arrays [52]. Processes responsible of identifying emotional saliency of the stimulus and of resolving cognitive conflicts appear to share these temporal properties [21, 79, 80]. There remains some debate regarding whether such an early activity would be informing about automatic or controlled mechanisms of attention [39].

On the other hand, in support to a potential automatic nature of the mechanisms sub-serving the interference effect observed during Threatening Flanker trials, we found slower responses to response-relevant targets when they were flanked by response-irrelevant Threatening images. However, our prediction that ERP modulations during early time widows would underpin such an effect was not supported by our data. We predicted that if the effect induced by Threatening Flankers does reflect automatic interference occurring during the orienting stages of disengaging attention, early modulation of relevant ERP activity (e.g., a P1-like component) would be observed [42, 37]. Instead, we found that ERP discrepancies over parietal and central-parietal regions in a late-time window seemingly accounted for such a behavioural pattern.

Threat-related stimuli seem to activate two complementary processes supported by the attentional network. Parra et al. [27] suggested that the fast-adaptive response to threat-related flankers is triggered by automatic mechanisms that can then activate top-down functions responsible for sustaining attention. The results presented here reveal that under the current stimulation conditions (i.e., supraliminal), the disengagement, shifting, and engagement of attention driven by threat-related flankers are seemingly underpinned by top-down processes. This does not rule out the possibility that such mechanisms are exogenously initiated (i.e., automatically) and endogenously kept (see Yiend, 2009 [16]). As noted before, people may be intrinsically motivated to attend to the relevant stimulus and try to get a glimpse of a nominally irrelevant, but somehow interesting (arousing) stimulus when time constraints allow to do so [25]. This is consistent with emotion related re-allocation of low-level attentional resources occurring after emotional cue extraction, which may not reflect a time-fixed shifting process [22]. Similar to the results reported by (Hickey, McDonald, & Theeuwes, 2006 [81]), our own results also demonstrate that participants tend to shift their attention to the target only after focusing on the more salient but task-irrelevant distractor. This pattern of results is in line with theories of attention in which stimulus-driven control plays an integral role. In the context of our paradigm, such endogenously driven processes could have been further facilitated by both the time allowed to process the stimuli (1000 ms) and the little interference posed by response relevant targets (i.e., line drawing of objects). This could explain why the timing of the behavioural and associated physiological responses linked to the emotional flanker effect was beyond our prediction, denoting later exogenous attentional effects compatible with those reported earlier (e.g., N2pc over parietal areas; [32, 81]). Future studies will need to investigate if the spatio-temporal dynamics here observed for the attentional bias to threat in the context of the Emotion Flanker Task holds when: i) response-irrelevant emotional IAPS stimuli presented as flankers compete with response-relevant emotional IAPS presented as targets; and ii) such a competition occurs under more constrained processing times (i.e., subliminal stimulus presentation).

We may suggest that the modulation observed within our earlier significant time window over central-parietal regions when Threatening targets were presented, and the modulation observed over the same regions within a late time window when Threatening flakers appeared may be indexing similar mechanisms. The former would be linked to endogenous processes quickly triggered to uptake information presented in the focus of attention, whereas the latter would reflect processes in charge of widely scanning the environment searching for potential threats when they are imminent, and the circumstances allow to do so. From this perspective, the attentional bias to threat could be a process exogenously triggered and endogenously kept [16].

The fact that we have observed such an attentional bias to threat under subliminal stimulus presentation conditions (200 ms) suggests that triggering such an adaptive response does not need long exposures. An alternative account could be that the sources of neural activity triggering the threat-related bias to response-irrelevant stimuli (e.g., fight-or-flight response) are too deep in the brain to be recorded via scalp EEG (e.g., amygdala, see [32, 82–84]). This fits well the suggestion about the automatic nature of this subtle yet adaptive response [83]. Of note, Carretié, 2014 [32] found that in 30.91% of the reviewed studies [17] neural activity, but not behavioural activity (which was also recorded), detected the attention bias toward emotional distractors. In line with recommendations from Carretié, 2014 [32] and from our own results, further studies using subliminal stimulus presentation and relying on ERP or fMRI methods will be need to investigate the temporal and spatial (i.e., cortical and subcortical candidates) dynamics of the attention bias to emotion.

It is striking that the function of such a sophisticated attentional system can be disrupted if response-irrelevant threat-related subliminal or supraliminal stimuli appear in the visual field outside the focus of attention [27, 85]. The saliency of threat-related flankers triggers seemingly automatic attentional mechanisms that appear to be involved in monitoring contextual information. Such mechanisms likely operate at a low detection threshold, which makes them highly sensitive to salient information that poses a potential threat [86]. Based on our current data, we can argue that early controlled attentional processes would allocate resources whenever the information presented in the focus of attention is arousing or potentially threatening. This would be followed by mechanisms responsible for extracting information and discriminating among stimuli that will engage a wider network. If during such processing an imminent threat appears in the environment, a wider attentional scanning would be launched. This further exploration would be neurally and behaviourally costly as it will hamper central attention by leaving less resources available, which will result in slower and less accurate responses.

There are some limitations to the present study which we would like to acknowledge. First, although we have previously found that response-key mappings would unlikely explain the patterns of results reported with the Emotional Flanker Task [27], future studies can implement counterbalancing procedures to further rule out the contribution of this factor. Second, although we took care of controlling the luminance level of IAPS images, such an approach was not used to select the stimuli but to explore their potential influence. Future studies will be needed to investigate the extent to which psychophysical properties of IAPS images (e.g., luminance, contrast, spatial frequency) could drive effects similar to those reported here, which have been long attributed to their emotional valences (see Lakens, Fockenberg, Lemmens, Ham, & Midden, 2013 [66]). Third, although Parra, Sanchez, Valencia, & Trujillo, 2017 [27] reported no effects of object category (i.e., living vs. non-living) on behavioural responses, future studies may add such stimuli to the screening test used within this paradigm to explore their true neutral value and also assess the extent to which such valance holds within the context of the Emotional Flanker Task (see for example Schneider, Veenstra, van Harreveld, Schwarz, & Koole, 2016 [87]). Finally, more research will be needed to further unveil the spatio-temporal dynamics of exogenous and endogenous attention processes supporting the adaptive response here investigated (i.e., attention bias to threat). To that aim, versions of the task enabling the assessment of congruency effects explored under subliminal stimulus presentation would add to the array of evidence so far accrued with this novel methodology.

## 5. Conclusions

The results presented in this study lead us to suggest that the attentional mechanisms involved in adaptive responses to threat-related stimuli seem to operate under different spatiotemporal dynamics. The saliency of threatening stimuli will bias attention whether they fall within or outside the focus of attention. The attention system spares resources that can be quickly deployed towards threat-relevant stimuli. Such a monitoring system operates through controlled processes responsible for extracting information from endogenously attended stimuli (i.e., targets) and spares resources to explore salient environmental signals through a feed-forward dynamic mechanism with potential survival value. This functional dynamic associated to threat detection grants our attentional system with an important evolutionary function [16, 88] and offers a valuable framework to further investigate the extent to which disruption of such processes could account for abnormal behaviours in developmental or neuropsychiatric disorders.

## Supporting information

S1 File Neurophysiological correlates of attentional bias to emotional stimuli. (DOCX)

S1 Data (XLSX)
