# Supplementary material for: Attentional bias during emotional processing: Behavioral and electrophysiological evidence from an Emotional Flanker Task
Source: PLoS One. 2021 Apr 2;16(4):e0249407. doi: 10.1371/journal.pone.0249407 (PMC8018632; doi:10.1371/journal.pone.0249407)
Supplement: S1 File — (DOCX) [file pone.0249407.s001.docx]

**S1 File**

**Neurophysiological correlates of attentional bias to emotional stimuli**

Natalia Trujillo, Diana Gómez, Sandra Trujillo, José David López, Agustín Ibáñez, Mario A Parra

**S1 Table.** Summary of the reviewed literature on attention bias to emotional stimuli using behavioral and EEG/ERP methodologies.

| **Paper** | **Target population and Sample size** | **Stimuli** | **Competition Model** | **Congruent /Incongruent Effect** | **Overt/Covert attention** | **Paradigm** | **Display Time (ms)** | **EEG Recording** | **EEG Activity/ERP Component** | **Key conclusions** |
| --- | --- | --- | --- | --- | --- | --- | --- | --- | --- | --- |
| 1. Seib-Pfeifer, L. E., Kirsten, H., & Gibbons, H. (2020). Attention please: ERP evidence for prime-target resource competition in the neutral-target variant of affective priming. *Acta Psychologica*, *208*, 103102. | University Student / N=44 | -Korean ideographs  -Photographs taken from the Open Affective Standardized Image Set (OASIS) | No | No | Overt/Covert attention. Target ideographs were preceded by visible prime pictures (negative, neutral or positive) that were introduced as irrelevant transitory stimuli. | Affective Priming (AP) paradigm | Presentation of a white fixation cross (random duration between 1000 and 1.500 ms) in the center of a black screen, followed by the prime 800 ms, and the ideographs until a response. | Yes | P1 (80-120ms)  P2 (180-230ms)  P300 (280-370ms)  PSW (460-700ms) | ERPs revealed reduced target processing when preceded by emotional primes compared to neutral ones. This was evidenced with components P2, P300, and PSW. This may suggest that the affective context influences the evaluative judgments measured with AP. |
| 2. Ásgeirsson, Á. G., & Nieuwenhuis, S. (2017). No arousal-biased competition in focused visuospatial attention. *Cognition*, *168*, 191-204. | People between 18 and 30 years of age  Experiment 1. N=28  Experiment 2. N=28  Experiment 3. N=21 | -Letters  -Exp. 1. IAPS pictures  -Exp. 2. Alerting tones  -Exp. 3. White-noise stimulation | Yes | No | Overt/Covert attention.  To identify a target presented with multiple distractors, which vary according to the degree of competence, considering the modeling of psychophysical data of the visual attention theory of Bundesen. | Arousal-biased competition theory in the context of focused visuospatial attention. | Exp. 1. A fixation cross for 4000ms, followed by an IAPS image for 1000ms. Then, a noise mask appeared for 500ms, before a second fixation period between 700 and 1300ms until the letter stimuli appeared, resulting in an interstimulus interval of 1200-1800ms.  Exp. 2. A fixation cross for 1000-3000 ms (jittered randomly to increase temporal uncertainty), followed by a letter stimulus. On the other half, a tone preceded the visual stimulus.  Exp. 3. A fixation cross constant for 500ms. An interstimulus interval for 5000ms for noise-induced arousal to return to baseline levels. | Yes | LPP (1200-1800 ms) | There was no evidence about arousal modulating the effect of distractor competition on the precision of target identification in any of the three experiments. The marked and prolonged higher LPP during IAPS images relative to noise mask and fixation period suggests that the images cause such modulation and that the physiological activation effect was still in progress at the time of the target presentation. |
| 3. He, M., Qi, C., Lu, Y., Song, A., Hayat, S. Z., & Xu, X. (2018). The sport expert’s attention superiority on skill-related scene dynamic by the activation of left medial frontal Gyrus: an ERP and LORETA study. *Neuroscience*, *379*, 93-102. | Varsity tennis players were recruited as experts /  N=19  Recreational tennis players were recruited as rookies / N=19 | -Pictures of five different sports (tennis, football, basketball, volleyball and baseball).  -Chinese Affective Picture System, faces of four negative emotions (anger, disgusting, sadness and fear).  -Pictures overlapping sport scene and negative face. | Yes | No | Overt/Covert attention.  Due to the overlap of stimuli. | Visual attention task, consisting of overlapping faces and scenes. | An overlapped image (S1) of 300 ms after a random Inter-stimulus interval (ISI) varying from 1400 to 2000 ms, either a sport scene or a negative emotional face (S2) overlapped in S1 was repeatedly presented for 300 ms. | Yes | The potentials induced by non-overlapped pictures (either a scene or negative face) were averaged and then subtracted the average from the potential induced by overlapped picture (scene and negative face). The search window for potential of attentional competition is 200–800 ms after stimulus onset. | The potential induced by tennis scene overlap was significantly greater than non-tennis overlap in experts, while in rookies it was absent. This suggests a superiority in attention associated with their abilities despite the distraction causing a negativity bias. |
| 4. Kennedy, B. L., Rawding, J., Most, S. B., & Hoffman, J. E. (2014). Emotion-induced blindness reflects competition at early and late processing stages: An ERP study. *Cognitive, Affective, & Behavioral Neuroscience*, *14*(4), 1485-1498. | Participants / N=26 | -IAPS pictures | No | No | Overt attention | Emotion-induced blindness (EIB). An image rotated 90° should be identified among a series of vertical images. | On each trial, participants viewed a sequence of 17 color pictures, with each image replacing the previous one every 100 ms. | Yes | N2 (185-275ms)  P3b (400-550ms) | Components N2 and P3b were found to be suppressed with irrelevant emotional distractors. These distractors elicited a positive deviation similar to the PD component associated with suppressing prominent and irrelevant distractors. This suggests that irrelevant emotional images gain access to working memory even when you try to ignore them. |
| 5. Deweese, M. M., Müller, M., & Keil, A. (2016). Extent and time‐course of competition in visual cortex between emotionally arousing distractors and a concurrent task. *European journal of neuroscience*, *43*(7), 961-970. | University Student / N=25 | -IAPS pictures  -Yellow dots | Yes | No | Overt/Covert attention | Coherent motion detection (dot) task | First, trigger 1000ms, followed scramble 1000ms, then scramble + dots 1750ms and finally dots + picture 6416ms. ISI from 3000-5000ms. | Yes | Steady-state visual evoked potential (SSVEP). | The higher amount of competition was evident in emotional distractors compared to neutrals. This suggests that the visual processing capacity measured with SSVEP is limited, resulting in priority processing to emotional content. |
| 6. Woody, M. L., Miskovic, V., Owens, M., James, K. M., Feurer, C., Sosoo, E. E., & Gibb, B. E. (2017). Competition effects in visual cortex between emotional distractors and a primary task in remitted depression. *Biological Psychiatry: Cognitive Neuroscience and Neuroimaging*, *2*(5), 396-403. | Population at high-risk for major depressive disorder (MDD) recurrence due to a recently remitted (rMDD) / N=27  Never-depressed women / N=28 | -Karolinska Directed Emotional Faces stimulus set (KDEF) displayed against a gray background.  -Gabor patch layer | No | No | Overt attention | A schematic layout of the change detection task, employing the frequency-tagging technique. | The facial stimulus was presented for 5000 ms. The intertrial interval was variable (2000-4000ms) | Yes | Steady-state visual evoked potential (SSVEP). | Women with rMDD show a deterioration in the attentional inhibition of emotional distractors, with a stronger effect on negative distractors (sad faces), regardless of their mood. This bias is stronger in those with less capacity in working memory. |
| 7. Heim, S., & Keil, A. (2019). Quantifying intermodal distraction by emotion during math performance: an electrophysiological approach. *Frontiers in Psychology*, *10*, 439. | University Student / N=20 | -Math problems  -Sound distractor | Yes | No | Overt/Covert attention | The Intermodal Distraction Task | It begins with a fixation point from 3000-5000ms, followed by three arithmetic problems each with a duration of 2000ms, while a distracting sound was activated in the free field throughout the period of 6000ms. | Yes | Steady-state visual evoked potential (SSVEP). | High-arousal sounds were associated with decreased visuo-cortical responses and poor performance, compared to low-arousal sounds and pink noise, suggesting that emotional distraction operates in all modalities. |
| 8. Boylan, M. R., Kelly, M. N., Thigpen, N. N., & Keil, A. (2019). Attention to a threat‐related feature does not interfere with concurrent attentive feature selection. *Psychophysiolog*, *56*(6), e13332. | University Student / N=16 | -Green and red dots  -Aversive loud noise | Yes | Yes | Overt/Covert attention | Aversive conditioning paradigms | All points were first presented on the screen with a random movement for 1749ms. Then, the onset of coherent motion occurred between 1750 and 6413ms, similar to the random segment. The coherent moving segment had a duration of 1749ms. Visual stimuli were presented for 8170ms, the interval between trials was 2500 to 3500ms. | Yes | Steady-state visual evoked potential (SSVEP). | When the threat signal was presented with the aversive noise, the SSVEP evoked by the threat signal was amplified. But this did not interact with the amplification of the task signal nor did it alter the interference visuo-cortical or behavioral observed during coherent movement. This suggests that the threat bias did not result in additional cost effects. |
| 9. Riels, K. M., Rocha, H. A., & Keil, A. (2020). No intermodal interference effects of threatening information during concurrent audiovisual stimulation. *Neuropsychologia*, *136*, 107283. | University Student / N=30 | Exp. 1.  -IAPS pictures  -Auditory tones  Exp. 2.  -IAPS pictures  -Auditory tones  -Aversive white noise | Yes | No | Overt/Covert attention | Rapid serial visual presentations (RSVPs) combined with auditory tones. | The tone lasted 6000 ms, starting 600 ms before the start of the images and 600 ms after the images were moved. Each image was presented for 150 ms. The ISI had a duration of 4000 to 4600 ms. | Yes | Steady-state visual evoked potential (SSVEP). | The visual stimulus condition had no significant effects on auditory sensory cortical responses in any of the experiments. The interference effects were more pronounced within the visual modality where a previously established destructive interference effect was clearly observed, regardless of the type and condition of the concurrent task. |
| 10. Ma, J., Liu, C., & Chen, X. (2016). Emotional modulation of conflict processing in the affective domain: evidence from event-related potentials and event-related spectral perturbation analysis. *Scientific Reports*, *6*, 31278. | University Student / N=27 | -Bodily Expression Action Stimulus Test  - Chinese affective picture system  -Chinese words (angry, sad and happy) | Yes | Yes | Overt/Covert attention | Emotional stroop task  Face-word  Body-word | A fixation screen for 500ms, followed by a blank screen ranging from 300-600ms. Then, the target stimulus for 1000 ms. Followed by the random ISI between 1200 and 1800 ms. | Yes | N1 (80-130 ms)  P2 (140-230 ms)  N450 (400-550 ms) | The face-word task evoked amplitudes N1 and P2 were greater than the body-word task. While the N450 had comparable amplitudes in both tasks. It was identified that in later stages of processing, the positive slow potentials were modulated by the emotion of the target and congruences. In addition, time-frequency analyzes evidenced improved theta band activity in the face-word task compared to the body-word task, both in early and later stages of processing. |
| 11. Schönwald, L. I., & Müller, M. M. (2014). Slow biasing of processing resources in early visual cortex is preceded by emotional cue extraction in emotion–attention competition. *Human Brain Mapping*, *35*(4), 1477-1490. | Participants / N=13 | -IAPS pictures  -Red squares | Yes | No | Overt/Covert attention | Foreground task with distracting background images to assess the processing of attentional competition in the visual cortex. | The time points were early (13% of trials, with a change in between 200 and 1,000 ms after trial onset), middle (60% of trials, 1,267–2,400 ms), or late (27% of trials, 2,467–4,333 ms). After each trial a blank screen with a red ‘‘x’’ in the middle was presented between 1,000 and 1,500 ms before the next trial started. | Yes | SSVEP (382-726ms)  EPN (190-359ms)  LPP (707-1035ms) | The ERPs showed an EPN associated with the identification of emotional signals. The LPP was related to a detailed processing of emotional images. The SSVEP was evoked by the foreground task. Regarding the emotional background images, the EPN and the LPP had a greater amplitude than the neutral ones, while the amplitudes of the SSVEP became significantly smaller. Additionally, no correlations were found among components, suggesting that they act independently. |
| 12. Li, Y., Xiao, X., Ma, W., Jiang, J., Qiu, J., & Zhang, Q. (2013). Electrophysiological evidence for emotional valence and competitive arousal effects on insight problem solving. *Brain research*, *1538*, 61-72. | University Student / N=36 | -Logogriphs. A logogriph may be a phrase, a Chinese proverb or a sentence in a poem.  -IAPS pictures | Yes | No | Overt/Covert attention | Learning-testing paradigm | A 2000 ms ready signal (“Ready?”), was followed by a fixation cross between 800 and 1200 ms at random. Subsequently, a positive or negative emotional picture was centered in the screen one at a time for 2000 ms. Finally, the picture was replaced by a blank screen (varied randomly from 400 to 800 ms), a test logogriph was then presented one at a time in the center of the screen and persisted until subjects solved the problem or a 4000 ms time limit reached. | Yes | The peak latencies and amplitudes of the N1 (120–180 ms) and P2 (220–280 ms) components as well as the mean amplitudes in the time window of 400–500, 800–1200, 1200–1600 and 1600–2500 ms. | The behavioral and ERPs results showed an increase in perception after negative emotional images in a competitive context. In this line, N1 and P1 were associated with an understanding of this context through unfocused attention. The N450 showed improved semantic integration and ease of attentional disengagement. The P800-1600 and P1600-2500 increased associations related to motivational arousal as a result of competition. This suggests that perception may be affected by emotional valence and the effects of competitive arousal. |
| 13. Voges, M. M., Giabbiconi, C. M., Gruber, T., Andersen, S. K., Hartmann, A. S., & Vocks, S. (2019). Sustained hypervigilance for one’s own body in women with weight and shape concerns: Competition effects in early visual processing investigated by steady-state visual evoked potentials (SSVEP). *Biological psychology*, *143*, 74-84. | Woman participants  Low body concern / N=24  High body concern / N=20 | Photographs of all participants, which were modified  -Slimmer version  -Overweight version  -Original version  -Scramble picture | Yes | No | Overt/Covert attention | Demanding dot detection task | First, scramble picture between 133 - 4400 ms, followed body picture between 133 - 4400 ms and an interstimulus interval between 900 - 1400 ms | Yes | Steady-state visual evoked potential (SSVEP). | A sustained reduction in the amplitude of the SSVEP is evidenced in the images of thin bodies or of average weight with respect to the images with overweight in both groups. Along the same lines, women with high body concerns had diminished amplitudes with images of their own bodies. Taken together, these results suggest patterns of covert surveillance and maintenance particularly in bodies that represent the slim ideal. Also, women with high body concerns show more attention to their own body that could intensify body dissatisfaction. |
| 14. Bekhtereva, V., & Müller, M. M. (2017). Bringing color to emotion: the influence of color on attentional bias to briefly presented emotional images. *Cognitive, Affective, & Behavioral Neuroscience*, *17*(5), 1028-1047. | Participants / N=27 | -IAPS pictures  -Light and dark gray dots | Yes | No | Overt/Covert attention | Visual detection task | The design began with the presentation of phase-coded images in a gray / color scale for 4533 ms. After a variable interval, the encoded image is changed to the original version for 133 ms and returns to its encoded form. | Yes | Steady-state visual evoked potential (SSVEP).  N1-EPN complex (170-400)  LPP (550-1000) | The findings identified that color images had a higher distracting effect than grayscale images, and this effect was intensified with unpleasant images. This was seen as evidenced by more pronounced early negativities in the N1-EPN complex. |
| 15. Müller, M. M., & Gundlach, C. (2017). Competition for attentional resources between low spatial frequency content of emotional images and a foreground task in early visual cortex. Psychophysiology, 54(3), 429-443. | Healthy young adults /  Experiment 1= 16  Experiment 2= 13  Experiment 3= 13 | IAPS images combined with moving red disks. For experiment three, IAPS images were selected based on its Low or High spatial frequency. | Yes | No | Covert | Distraction paradigm | Trials started with a fixation point. Then, the point of change was defined and pseudorandomized for three time windows: early (200-1000ms), middle (1267-2400ms) and late (2467-4333ms). After each trial blank screen with a red x was presented for 1000-1500ms.  For experiment two, the exposure time was 2000ms.  For experiment three, images were presented for 1300ms. | Yes | Steady-state visual evoke potential, P1 (115-135ms)  N1-EPN (200-400ms)  LPP (500-1000ms) | Low-spatial frequency allows cue extraction for emotional recognition, but only when such images were presented without competing stimuli.  In addition, for the LPP the main effect was differential emotional amplitude in high-spatial frequency images. |
| 16. Schupp, H. T., Schmälzle, R., & Flaisch, T. (2014). Explicit semantic stimulus categorization interferes with implicit emotion processing. Social cognitive and affective neuroscience, 9(11), 1738-1745. | University students N=24 | IAPS images (pleasant, unpleasant and neutral) | Yes | No | Overt | Letter, shape and orientation categorization/discrimination  task | For active task, picture presentation time was 26.6ms followed by an inter-stimulus interval of 750-1250 ms, in which participants had to respond. For passive task, the time window for the trial was identical. | Yes | Temporo-Occipital negativity (200-300ms)  LPP (300-600ms) | Results indicate that explicit semantic information interfere with covert/implicit analysis during the competition for sharing resources. In addition, passive viewing condition described significant ERPs differences among emotional vs. neutral conditions. Such effect disappearance in active view. Authors suggest that this effect is produced by attentional competition. |
| 17. Lv, J. Y., Wang, T., Tu, S., Zheng, F., & Qiu, J. (2011). The effect of different negative emotional context on involuntary attention: an ERP study. Brain research bulletin, 86(1-2), 106-109. | University students/ N=12 | Emotional and irrelevant standard images and environmental sounds. | Yes | No | Both | Emotional task using relevant and irrelevant conditions. | Fixation cross was presented for 300ms, followed by emotional image presentation (300 ms) and a sound (200ms). Total trial time was 1800ms. | Yes | MMN (150-250ms)  Novelty-P3 (250-400ms) | Early involuntary attention modulation represented by the MMN was not affected by emotional context. For late modulation, it was shorter for sound conditions when negative valences were presented. |
| 18. Watts, S., Buratto, L. G., Brotherhood, E. V., Barnacle, G. E., & Schaefer, A. (2014). The neural fate of neutral information in emotion‐enhanced memory. Psychophysiology, 51(7), 673-684. | Healthy adults / N=34 | IAPS and Google images | Yes | No | Covert/memory encoding | Recall task | A fixation cross was presented in the center of the screen for 600 ms, followed by neutral or emotional images for 1500 ms. | Yes | Window 1= 200-400ms  Window 2= 400-800ms  Window 3= 800- 1500ms | ERP analysis showed that the recall process was lower during neutral and intermixed trials than for other conditions. The authors suggested that such effect is a result of the asymmetrical attentional and working memory competition. |
| 19. MacNamara, A., Ferri, J., & Hajcak, G. (2011). Working memory load reduces the late positive potential and this effect is attenuated with increasing anxiety. Cognitive, Affective, & Behavioral Neuroscience, 11(3), 321-331. | University students / N=47 | IAPS | Yes | No | Overt-Recall | Letter recall task | Authors present an array of letters for 5000ms, followed by a fixation cross for 500 -1000ms. Then, an emotional image was presented for 2000ms. The task ask for the letter´s recall in the presence of emotional stimuli. | Yes | LPP (400-100ms and 1000-2000ms) | LPP was larger over low-load (e.g. neutral valence) trials in comparison with high-load (e.g. aversive valence). This study suggest that emotional content produces a distracting effect and constrain working memory recall among subjects with different levels of anxiety. |
| 20. Bekhtereva, V., Craddock, M., & Müller, M. M. (2015). Attentional bias to affective faces and complex IAPS images in early visual cortex follows emotional cue extraction. Neuroimage, 112, 254-266. | Healthy adults / N= 24 | Faces and images | Yes | No | Covert | Distraction paradigm | Trials started with a fixation point. Then, the point of change was defined and pseudorandomized for three time-windows: early (200-1000 ms), middle (1267-2400ms) and late (2467-4333ms). | Yes | Steady-state visual evoked potentials (SSVEPs)  P1(face 110-130ms and IAPS 110 to 190 ms) N170 (face 156 to 196 ms)  N1 (IAPS 202-242 ms)  EPN (IAPS 276-426 ms) | Authors found that the emotional IAPS irrelevant task subtracts electrophysiological resources for the response of the foreground task. Furthermore, the modulation of SSVEP was observed earlier for faces (around 180 ms) than for IAPS pictures (around 400 ms). |
| 21. Wieser, M. J., McTeague, L. M., & Keil, A. (2012). Competition effects of threatening faces in social anxiety. Emotion, 12(5), 1050. | University student/ N= 34 (from 849 that completed the screening) 17 with low and 17 with high anxiety.  . | Angry, happy and neutral faces and black-and-white sinusoidal gratings | Yes | No | Overt | Task-irrelevant facial expressions and Task-relevant  Gabor patch stream / change detection paradigm | In each experimental trial, a face picture was presented at the center of the screen for 3000 ms. | Yes | SSVEP | Threatening faces generate a larger competition effect represented by a large amplitude for the SSVEP among individual with social anxiety.  For task relevant Garbor grating was diminished for threatening images in comparison to neutral and happy faces distractors. |
| 22. Wieser, M. J., & Keil, A. (2011). Temporal trade-off effects in sustained attention: dynamics in visual cortex predict the target detection performance during distraction. Journal of Neuroscience, 31(21), 7784-7790. | University students/  Experiment 1= 19  Experiment 2= 16 | Angry, happy and neutral faces and IAPS images. Black-and-white sinusoidal gratings | Yes | No | Overt | Task-irrelevant facial expressions and Task-relevant  Gabor patch stream/ change detection paradigm | In each  experimental trial, a face picture was presented at the center of the screen for 3000 ms. | Yes | SSVEP | The early overallocation of the visual cortex was associated with reduction in electrophysiological activity and reduced change detection. The results support the model that sustained attention is related to limited resources competition. |
| 23. Miskovic, V., & Keil, A. (2013). Perceiving threat in the face of safety: excitation and inhibition of conditioned fear in human visual cortex. Journal of Neuroscience, 33(1), 72-78. | University students / N=29 | black-and-white sinusoidal gratings and sound cues | Yes | No | Covert | Discrimination paradigm | A fixation cross was presented in the center of the screen for 1000ms and stay visible for the rest of the trial (5700-6900ms).  For the non-reinforced conditioning trials, overall trial time was 5700ms and for the reinforced conditioning trials was 6900ms length. Trials additionally included an extra 1200ms for the former unconditioned stimulus. | Yes | SSVEP | Authors inform a facilitation for threatening cues perception in comparison with those representing safety and novel cues. The key finding suggest that perceptual biases are crucial for the acquisition of danger and safety signal. |
| 24. Müller, M. M., Andersen, S. K., & Attar, C. H. (2011). Attentional bias to briefly presented emotional distractors follows a slow time course in visual cortex. Journal of Neuroscience, 31(44), 15914-15918. | Health adults / N=20 | Emotional and neutral IAPS images | Yes | No | Covert | Target detection task | Each trial lasted for 4100 ms. It initially presented a scramble IAPS image for a variable period, followed by its concrete version for 200ms and then back to the former scramble image. | Yes | SSVEP | Authors found that brief presentation of emotional distractors trigger attentional bias. This study informs a potential slow feedback neural competition mechanism for emotional distractors that continues after the end of the emotional stimulus. The ERP amplitude was greater for emotional images with respect to neutral. |
| 25. Deweese, M. M., Bradley, M. M., Lang, P. J., Andersen, S. K., Müller, M. M., & Keil, A. (2014). Snake fearfulness is associated with sustained competitive biases to visual snake features: hypervigilance without avoidance. Psychiatry research, 219(2), 329-335. | University student / from 561 that response the pre-screening N=41 (all females) were selected. Sample selected described different levels of snake fear. | IAPS images (e.g. pleasant-kitten, neutral-cow and unpleasant-snake) | Yes | No | Covert | Target detection task | The total trial time was 9715ms. The interval for the coherent motion was between 1170 to 7000ms plus a variable 3000 to 5000ms intertrial interval. | Yes | SSVEP | Authors informed that emotionally irrelevant task subtract important attentional resources from the relevant competitors, interfering with the visual processing of a concurrent task stimulus. |
| 26. West, G. L., Anderson, A. A., Ferber, S., & Pratt, J. (2011). Electrophysiological evidence for biased competition in V1 for fear expressions. Journal of Cognitive Neuroscience, 23(11), 3410-3418. | University students/  Experiment 1= 10  Experiment 2= 12  Experiment 3= 10 | Fear and neutral faces | Yes | No | Covert | Distraction paradigm | Fixation cross was presented for 600-1000 ms followed by the layout including fearful or neutral images for 100 ms and a blank screen until the response. | Yes | C1 (0-200ms) | The ERP maximum amplitude modulation was observed around 50ms biased in favor of fear expressions in comparison with neutrals. In addition, authors informed that emotional content instead of low level features is associated with V1 modulation. Finally, authors suggest that the competition for attentional resources started before V1 encoding. |
| 27. Junhong, H., Renlai, Z., & Senqi, H. (2013). Effects on automatic attention due to exposure to pictures of emotional faces while performing Chinese word judgment tasks. PLoS One, 8(10), e75386. | Undergrad and graduated university students /  Experiment 1 N=35  Experiment 2 N=26 | Emotional facial expression (fearful, neutral and happy) | Yes | No | Overt and covert | Low or high demand cognitive tasks under unattended emotional facial categorization conditions. | A fixation cross was centrally presented for 500 ms, followed by a Chinese word for 100ms together with an identical emotionally charged human face presented in both sizes of the Chinese word.  After 1900 ms a blank screen is showing asking for individual’s response for 850ms. | Yes | P2 (154- 190ms) | Unattended fearful faces require more attentional resources than unattended neutral faces on a low cognitive load task; however, this effect was not observed on a high cognitive load task. Authors conclude that fearful faces might automatically capture attention if some of the attentional resources were available under the unattended condition. |
| 28. Bertsch, K., Böhnke, R., Kruk, M. R., Richter, S., & Naumann, E. (2011). Exogenous cortisol facilitates responses to social threat under high provocation. Hormones and behavior, 59(4), 428-434. | University students / N=56 | Three male and three female faces showing happy, angry, fearful,  and neutral expressions were taken from Ekman and Matsumoto's  Japanese and Caucasian Facial Expressions of Emotion (JACFEE) and Japanese and Caucasian Neutral Faces (JACNeuF) (Matsumoto and  Ekman, 1988). | Yes | Yes | Covert attention-stroop effect | Manipulation of acute cortisol level. Individuals were exposed to a competitive and aggression paradigm by using emotional faces.  Emotional stroop task. | A fixation cross was presented for 1990ms, followed by colored facial expression stimuli for 16.6ms that is replaced by a color mask until individuals respond. | Yes | P1 (100-170ms)  P2 (180-220ms)  LPP (600-800ms) | The enhancing in early and late positive ERP component could be associated with increase in the available social information. On the other hand, cortisol trigger a reduction in the early frontocentral bias for angry faces. |

**S2 Table.** Descriptive statistics of trials by condition

|  | | | |  |
| --- | --- | --- | --- | --- |
| Condition | N | M (SD) | LL | UL |
| TC | 32 | 49.03 (13.58) | 44.13 | 53,93 |
| TP | 32 | 49.03 (11.12) | 45.02 | 53.04 |
| NC | 32 | 41.47 (13.58) | 36.57 | 46.37 |
| NP | 32 | 47.28 (10.32) | 45,87 | 53.31 |

*Note.* Threat Central (TC); Threat Peripheral (TP); Neutral Central (NC); Neutral Peripheral (NP); Lower Limit (LL); Upper Limit (UL).

The results of the contingency table of the frequency distribution analyzed from a chi-square (X^2^) did not show significant differences between the conditions (X^2^ = 0.22, p = 0.64).

| **S3 Table.** Correlation Matrix of the behavior by regions of interest in the condition Threat Central | | | | | | | | | | | | | |
| --- | --- | --- | --- | --- | --- | --- | --- | --- | --- | --- | --- | --- | --- |
|  | |  | | RT_TC | | ROI1_TC | | ROI2_TC | | ROI3_TC | | ROI4_TC | |
| RT_TC |  | Pearson's r |  | — |  |  |  |  |  |  |  |  |  |
|  |  | p-value |  | — |  |  |  |  |  |  |  |  |  |
| ROI1_TC |  | Pearson's r |  | 0.481 |  | — |  |  |  |  |  |  |  |
|  |  | p-value |  | 0.005 |  | — |  |  |  |  |  |  |  |
| ROI2_TC |  | Pearson's r |  | 0.187 |  | 0.596 |  | — |  |  |  |  |  |
|  |  | p-value |  | 0.304 |  | < .001 |  | — |  |  |  |  |  |
| ROI3_TC |  | Pearson's r |  | 0.213 |  | 0.520 |  | 0.851 |  | — |  |  |  |
|  |  | p-value |  | 0.241 |  | 0.002 |  | < .001 |  | — |  |  |  |
| ROI4_TC |  | Pearson's r |  | 0.266 |  | 0.563 |  | 0.683 |  | 0.701 |  | — |  |
|  |  | p-value |  | 0.142 |  | < .001 |  | < .001 |  | < .001 |  | — |  |

*Note.* Reaction Time (RT); Threat Central (TC); Parietal and central-parietal regions in a time window 300 – 400ms (ROI-1); Parietal and central-parietal regions in a time window 550 – 690ms (ROI-2); Parietal and parietal-occipital regions in a time window 500 – 700ms (ROI-3); Frontal regions in a time window 500 – 700ms (ROI-4).

| **S4 Table.** Correlation Matrix of the behavior by regions of interest in the condition Threat Peripheral | | | | | | | | | | | | | |
| --- | --- | --- | --- | --- | --- | --- | --- | --- | --- | --- | --- | --- | --- |
|  | |  | | RT_TP | | ROI1_TP | | ROI2_TP | | ROI3_TP | | ROI4_TP | |
| RT_TP |  | Pearson's r |  | — |  |  |  |  |  |  |  |  |  |
|  |  | p-value |  | — |  |  |  |  |  |  |  |  |  |
| ROI1_TP |  | Pearson's r |  | 0.098 |  | — |  |  |  |  |  |  |  |
|  |  | p-value |  | 0.595 |  | — |  |  |  |  |  |  |  |
| ROI2_TP |  | Pearson's r |  | -0.104 |  | 0.477 |  | — |  |  |  |  |  |
|  |  | p-value |  | 0.571 |  | 0.006 |  | — |  |  |  |  |  |
| ROI3_TP |  | Pearson's r |  | 0.100 |  | 0.477 |  | 0.838 |  | — |  |  |  |
|  |  | p-value |  | 0.586 |  | 0.006 |  | < .001 |  | — |  |  |  |
| ROI4_TP |  | Pearson's r |  | 0.129 |  | 0.323 |  | 0.690 |  | 0.770 |  | — |  |
|  |  | p-value |  | 0.482 |  | 0.072 |  | < .001 |  | < .001 |  | — |  |
| *Note.* Reaction Time (RT); Threat Peripheral (TP); Parietal and central-parietal regions in a time window 300 – 400ms (ROI-1); Parietal and central-parietal regions in a time window 550 – 690ms (ROI-2); Parietal and parietal-occipital regions in a time window 500 – 700ms (ROI-3); Frontal regions in a time window 500 – 700ms (ROI-4).   \| **S5 Table.** Correlation Matrix of the behavior by regions of interest in the condition Neutral Central \| \| \| \| \| \| \| \| \| \| \| \| \| \| \| --- \| --- \| --- \| --- \| --- \| --- \| --- \| --- \| --- \| --- \| --- \| --- \| --- \| --- \| \|  \| \|  \| \| RT_NC \| \| ROI1_NC \| \| ROI2_NC \| \| ROI3_NC \| \| ROI4_NC \| \| \| RT_NC \|  \| Pearson's r \|  \| — \|  \|  \|  \|  \|  \|  \|  \|  \|  \| \|  \|  \| p-value \|  \| — \|  \|  \|  \|  \|  \|  \|  \|  \|  \| \| ROI1_NC \|  \| Pearson's r \|  \| 0.174 \|  \| — \|  \|  \|  \|  \|  \|  \|  \| \|  \|  \| p-value \|  \| 0.340 \|  \| — \|  \|  \|  \|  \|  \|  \|  \| \| ROI2_NC \|  \| Pearson's r \|  \| 0.205 \|  \| 0.180 \|  \| — \|  \|  \|  \|  \|  \| \|  \|  \| p-value \|  \| 0.259 \|  \| 0.326 \|  \| — \|  \|  \|  \|  \|  \| \| ROI3_NC \|  \| Pearson's r \|  \| 0.054 \|  \| -0.024 \|  \| 0.548 \|  \| — \|  \|  \|  \| \|  \|  \| p-value \|  \| 0.771 \|  \| 0.896 \|  \| 0.001 \|  \| — \|  \|  \|  \| \| ROI4_NC \|  \| Pearson's r \|  \| 0.302 \|  \| -0.028 \|  \| 0.662 \|  \| 0.502 \|  \| — \|  \| \|  \|  \| p-value \|  \| 0.093 \|  \| 0.880 \|  \| < .001 \|  \| 0.003 \|  \| — \|  \| | | | | | | | | | | | | | |

| *Note.* Reaction Time (RT); Neutral Central (NC); Parietal and central-parietal regions in a time window 300 – 400ms (ROI-1); Parietal and central-parietal regions in a time window 550 – 690ms (ROI-2); Parietal and parietal-occipital regions in a time window 500 – 700ms (ROI-3); Frontal regions in a time window 500 – 700ms (ROI-4).  **S6 Table.** Correlation Matrix of the behavior by regions of interest in the condition Neutral Peripheral | | | | | | | | | | | | | |
| --- | --- | --- | --- | --- | --- | --- | --- | --- | --- | --- | --- | --- | --- |
|  | |  | | RT_NP | | ROI1_NP | | ROI2_NP | | ROI3_NP | | ROI4_NP | |
| RT_NP |  | Pearson's r |  | — |  |  |  |  |  |  |  |  |  |
|  |  | p-value |  | — |  |  |  |  |  |  |  |  |  |
| ROI1_NP |  | Pearson's r |  | -0.026 |  | — |  |  |  |  |  |  |  |
|  |  | p-value |  | 0.889 |  | — |  |  |  |  |  |  |  |
| ROI2_NP |  | Pearson's r |  | -0.047 |  | 0.286 |  | — |  |  |  |  |  |
|  |  | p-value |  | 0.797 |  | 0.113 |  | — |  |  |  |  |  |
| ROI3_NP |  | Pearson's r |  | 0.185 |  | 0.197 |  | 0.856 |  | — |  |  |  |
|  |  | p-value |  | 0.310 |  | 0.281 |  | < .001 |  | — |  |  |  |
| ROI4_NP |  | Pearson's r |  | 0.045 |  | 0.187 |  | 0.782 |  | 0.759 |  | — |  |
|  |  | p-value |  | 0.808 |  | 0.305 |  | < .001 |  | < .001 |  | — |  |

*Note.* Reaction Time (RT); Neutral Peripheral (NP); Parietal and central-parietal regions in a time window 300 – 400ms (ROI-1); Parietal and central-parietal regions in a time window 550 – 690ms (ROI-2); Parietal and parietal-occipital regions in a time window 500 – 700ms (ROI-3); Frontal regions in a time window 500 – 700ms (ROI-4).

**S7 Table.** Mean score (from 1 to 5) for each emotional valence allocated by research participants during emotional screening given after the Emotional Flanker Task.

| **Neutral Images** | | | | **Threatening Images** | | | |
| --- | --- | --- | --- | --- | --- | --- | --- |
| **Rated as Neutral** | | **Rated as Threatening** | | **Rated as Neutral** | | **Rated as Threatening** | |
| Mean | SD | Mean | SD | Mean | SD | Mean | SD |
| 4.30 | 0.79 | 1.52 | 0.63 | 1.40 | 0.59 | 4.21 | 0.70 |

The scores show a high consistency between the perceived and actual valences of the images used in this task.
